# Aortic Valve Embryology, Mechanobiology, and Second Messenger Pathways: Implications for Clinical Practice

**DOI:** 10.3390/jcdd11020049

**Published:** 2024-02-01

**Authors:** Maximiliaan L. Notenboom, Lucas Van Hoof, Art Schuermans, Johanna J. M. Takkenberg, Filip R. Rega, Yannick J. H. J. Taverne

**Affiliations:** 1 Department of Cardiothoracic Surgery, Erasmus University Medical Center, 3000 CA Rotterdam, The Netherlands; m.l.notenboom@erasmusmc.nl (M.L.N.);; 2 Department of Cardiac Surgery, University Hospitals Leuven, 3000 Leuven, Belgium

**Keywords:** aortic valve disease, embryology, hemodynamics, second messenger pathways

## Abstract

During the Renaissance, Leonardo Da Vinci was the first person to successfully detail the anatomy of the aortic root and its adjacent structures. Ever since, novel insights into morphology, function, and their interplay have accumulated, resulting in advanced knowledge on the complex functional characteristics of the aortic valve (AV) and root. This has shifted our vision from the AV as being a static structure towards that of a dynamic interconnected apparatus within the aortic root as a functional unit, exhibiting a complex interplay with adjacent structures via both humoral and mechanical stimuli. This paradigm shift has stimulated surgical treatment strategies of valvular disease that seek to recapitulate healthy AV function, whereby AV disease can no longer be seen as an isolated morphological pathology which needs to be replaced. As prostheses still cannot reproduce the complexity of human nature, treatment of diseased AVs, whether stenotic or insufficient, has tremendously evolved, with a similar shift towards treatments options that are more hemodynamically centered, such as the Ross procedure and valve-conserving surgery. Native AV and root components allow for an efficient Venturi effect over the valve to allow for optimal opening during the cardiac cycle, while also alleviating the left ventricle. Next to that, several receptors are present on native AV leaflets, enabling messenger pathways based on their interaction with blood and other shear-stress-related stimuli. Many of these physiological and hemodynamical processes are under-acknowledged but may hold important clues for innovative treatment strategies, or as potential novel targets for therapeutic agents that halt or reverse the process of valve degeneration. A structured overview of these pathways and their implications for cardiothoracic surgeons and cardiologists is lacking. As such, we provide an overview on embryology, hemodynamics, and messenger pathways of the healthy and diseased AV and its implications for clinical practice, by relating this knowledge to current treatment alternatives and clinical decision making.

## 1. Introduction

Heart valve disease stands for a major worldwide burden and is associated with substantial mortality and morbidity [1]. Absolute numbers of deaths attributed to aortic valve disease (AVD) have increased over the past 30 years [2], making AVD responsible for the largest proportion of deaths within the spectrum of valvular heart disorders. When left untreated, the natural course of AVD, especially aortic valve stenosis (AS), is progressive [3,4] and associated with high mortality rates across the entire spectrum [5].

Taking a deeper look into the process of valve degeneration, this appears to be a multifactorial process which includes embryological, genetic, hemodynamic, structural, and cellular factors [6]. Importantly, healthy aortic valve (AV) leaflets consist of multiple layers which communicate with surrounding tissues through humoral [7,8] and mechanical stimuli [9]. Neurofilaments, (myo)fibroblasts, endothelial cells, extracellular matrix (ECM) components, and even smooth muscle cells (SMCs) play crucial roles in these regulatory processes [7], which are adaptive to shear stress (mechanotransduction) and blood compositions (humoral). Chronic or acute disturbance of an AV’s homeostatic state may induce AS and/or aortic valve regurgitation (AR) through various mechanisms [10]. More than just the visible, clinically documentable structural defects to the AV, which are measured using current routine clinical imaging techniques, are associated with AV dysfunction; it is acknowledged that pathophysiology induces morphologic changes. Messenger pathways that are disturbed by altered flow dynamics or humoral stimuli may in itself induce an effect on a functional level [7,11]. Importantly, the AV is known to generate contractile, secretory, and proliferative responses to these stimuli [7,8,9,12] and the interplay between physics and biology as well as the interplay between form and function play essential roles.

Treatment of AVD is commonly limited to prosthetic AV replacement (AVR). However, insights into the benefits of preserving one’s native valve and functional abilities have led to an expansion of the surgical armamentarium by development of innovative techniques, including the Ross procedure [13] and AV repair techniques [14]. A perfect solution does not exist, and the preferred treatment for an individual with AVD should be patient-tailored. But understanding the healthy and diseased AV on a cellular and biomechanical level is essential in appreciating the benefits of particular treatment options. The aim of this review is to provide an overview of the embryology, genetics, structure, pathophysiology, messenger pathways, and hemodynamics of the AV, while linking these to their implications for treatment.

## 2. Embryology of the Aortic Valve

### Normal Outflow Tract and Valve Formation

Twenty–thirty percent of congenital cardiovascular malformations contain some form of defective heart valves. Its incidence has been estimated to be as high as 5% of live births [15]. Several seminal papers have been published on the different steps of cardiac development [16,17,18]; within the scope of this paper, we will solely focus on the embryogenesis of the heart valves.

The formation of endocardial cushions in the atrioventricular canal (AVC) and outflow tract (OFT) marks the start of valvulogenesis in the primitive looped heart at approximately 31–35 days after conception [19,20]. Contrary to the endocardial cushion and valvular leaflet relationship in the AVC, less is known about how the semilunar valves arise from the complex arrangement of the endocardial cushions in the OFT [15]. Opposing dextro-superior and sinistro-inferior endocardial cushions grow at the cephalad portion of the truncus arteriosus [21]. Simultaneously to the creation of these conotruncal cushions, two intercalated cushions form in between the aforementioned cushions. Upon fusion of the conotruncal cushions, the truncal septum is formed. At the early developmental stages, those conotruncal cushions appear bulky and cellularized, because the endothelial cells overlying the primitive endocardial cushions invade the conotruncal cushion matrix [22,23]. This highly proliferative state of endocardial cushions is lost in later remodeling and mature valves [15,22]. The growth of these valvular primordia continues, leading to the formation of thin fibrous cusps for the semilunar valves until they have become highly organized structures containing a rich collagen, proteoglycan, and elastin ECM by the end of gestation [22]. Valve maturation and microarchitectural remodeling will continue into juvenile stages in all mammalian species [15,22,24] with similar stratification patterns between species [22]. The evolutionary basis for this similar semilunar valvulogenesis throughout mammalian species resides on highly preserved molecular pathways and physiological processes that were present in species with tubular hearts driving unidirectional flow and have been maintained throughout the formation of a four-chambered heart [17].

**Figure 1:**
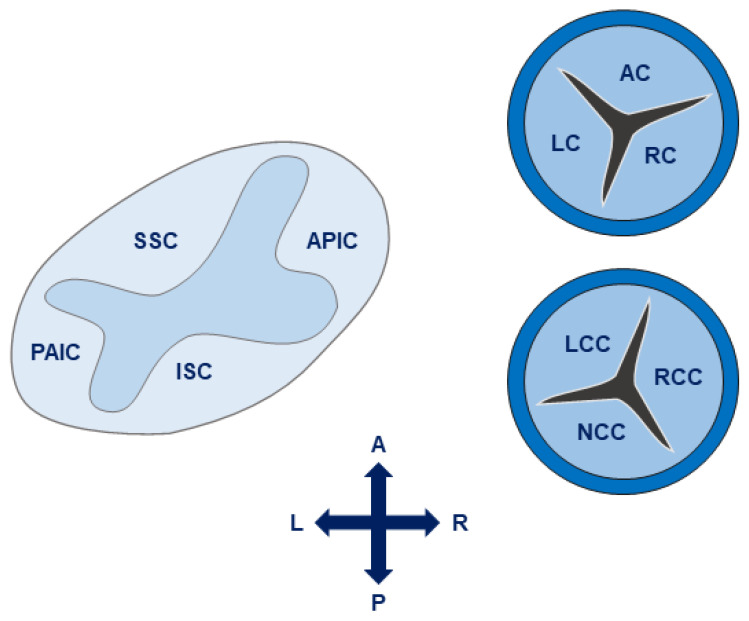
Leaflet development of the semilunar valves (aortic and pulmonary). The left and right valve leaflets of both the pulmonary and aortic valve arise from the conotruncal cushions, whereas the non-coronary aortic leaflet and anterior pulmonic leaflet arise from the intercalated cushions. Adapted from Martin et al., 2015 [23], distributed under the terms and conditions of the Creative Commons Attribution license (CC BY 4.0 DEED, Attribution 4.0 International), 2015, the authors.

**Figure 2:**
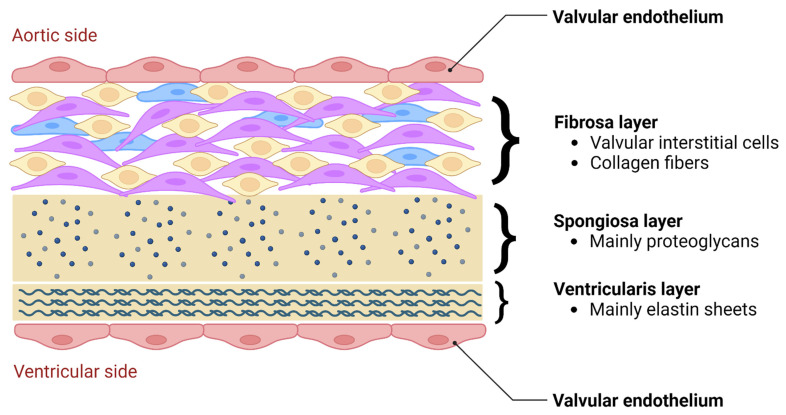
Microarchitectural composition of aortic valve leaflets, consisting of a bilayer of endothelial cells (aortic and ventricular side), fibrosa layer, spongiosa layer, and ventricularis layer.

During truncal septal differentiation with conotruncal rotation and caudal shift, mesenchymal derivation from the endocardium takes place [25], where dedifferentiation from a myosin-heavy chain to an alpha-smooth muscle actin phenotype takes place. This allows for the formation of (mature) semilunar valves from the conotruncal and intercalated cushions of the OFT (Figure 1) [23,25]. The conotruncal cushions give rise to the semilunar right and left leaflets; that is, the right and left coronary cusp leaflets for the aorta. The opposing right and left intercalated cushions develop into the posterior aortic (non-coronary cusp) and anterior pulmonic leaflet, respectively [23]. During endocardial cushion fusion, cavities are formed through apoptosis leading to the formation of a central lumen of each of the cushions separating the three valves and creating the wall of the supporting sinuses through peripheral arterialization [23,26]. During this process of cavitation the muscular portion of the proximal OFT contributes to the formation of valves and sinuses in a paracrine, rather than a direct cellular fashion [23]. Eventually, rudimentary valves will elongate and the endocardial cushions thin out, thus remodeling the valves with a compartmentalized microarchitecture consisting of five layers: endothelium, fibrosa (comprising mainly valvular interstitial cells (VICs) and collagen fibers), spongiosa (comprising mainly proteoglycans), ventricularis (comprising mainly elastin sheets), and endothelium [22] (Figure 2). The microarchitectural composition of the valve leaflets together with the biomechanical properties and vasoactivity are critical for normal valve function allowing for optimal interplay between form and function. A striking example of this interplay is the occurrence of hypoplastic left heart syndrome during heart development, where the obstruction or atresia of left-sided valves produces a lack of blood flow, in turn affecting the functional stimuli–i.e., flow–to form a compact left ventricle (LV) [27,28]. Such findings emphasize the role of blood flow perturbations as a causal factor in, not only early organogenesis, but also pathogenesis of valvular (structural) disease.

## 3. Transcriptional Regulation of Valvulogenesis

**Figure 3:**
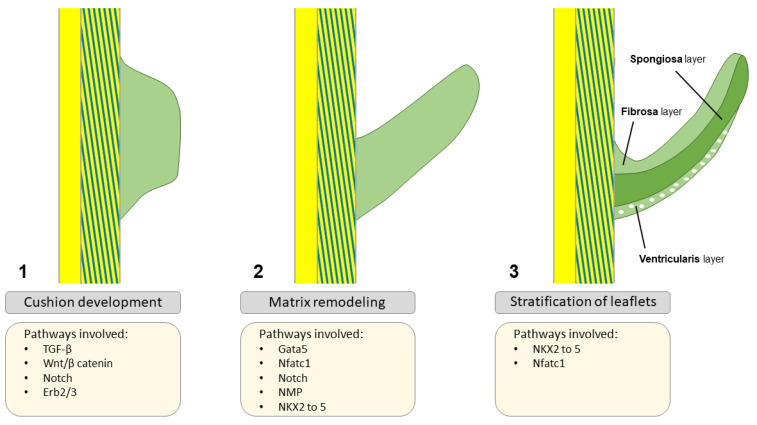
Aortic valve formation: starting with cushion formation (1), proceeding with matrix remodeling (2), and ending with leaflet stratification (3). Adapted from Martin et al., 2015 [23], distributed under the terms and conditions of the Creative Commons Attribution license (CC BY 4.0 DEED, Attribution 4.0 International), 2015, the authors.

**Figure 4:**
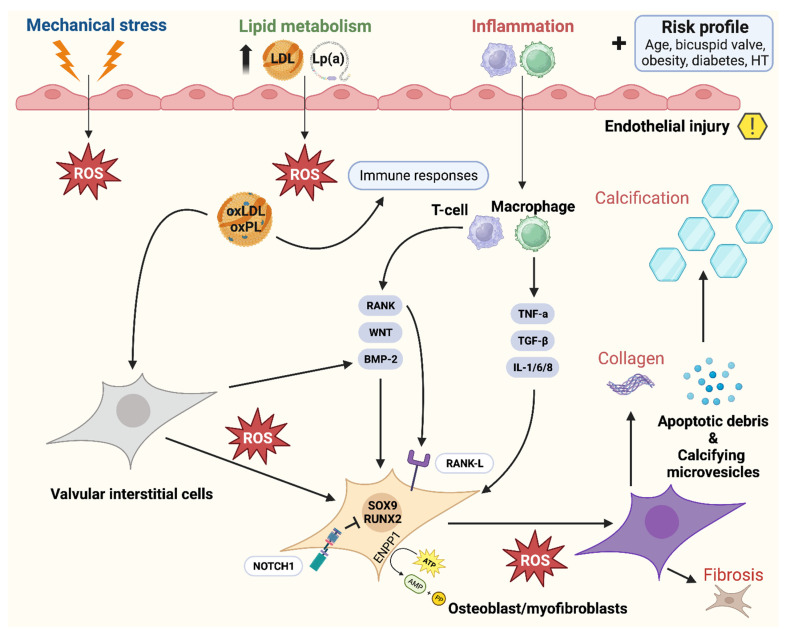
Pathophysiology of calcific aortic valve disease: activation of bone-formation pathways induces aortic valve calcification through various ligands, ranging from inflammation to metabolism. Adapted from Greenberg et al. [29], distributed under the terms of the Creative Commons CC BY license, 2021, the authors. Legend: Calcific aortic valve disease is initiated by endothelial injury (right upper quadrant), and propagated by a complex cascade of signaling involving osteoblasts and myofibroblasts (bone formation pathways). Abbreviations (alphabetical): ATP = adenosine triphosphate; AMP = adenosine monophosphate; BAV = bicuspid aortic valve; BMP2 = bone morphogenic protein 2; ENPP1 = ectonucleotide pyro phosphatase/phosphodiesterase family member 1; IL = Interleukin; LDL = low-density lipoprotein; Lp(a) = lipoprotein a; ROS = reactive oxygen species; NOTCH1 = Notch homolog 1; PP = inorganic pyrophosphate; RUNX2 = runt-related transcription factor 2; SOX9 = SRY-box 9; TGF-β = transforming growth factor beta; TNF = tumor necrosis factor alpha.

Normal development and function require tightly regulated interactions between molecules and gene transcription, and any genetic defect or signaling alteration may disturb this process, leading to structural malformations. There is a complex genetic regulatory network that generates valve progenitor cells through endothelial-to-mesenchymal transformation (EMT), effectuates ECM remodeling, and is involved in leaflet stratification (Figure 3). Importantly, calcific aortic valve disease (CAVD), a progressive condition often stimulated by inflammatory processes—in which a tricuspid AV or a congenital bicuspid AV (BAV) becomes thickened, fibrosed, and, consequently, calcified [29]—is characterized by the expression of transcription factors also involved in valve development and comparable to osteogenesis [21]. Moreover, mechanosensitive systems, e.g., RhoA/ROCK and YAP/TAZ, detect substrate changes and initiate mineralization pathways leading to CAVD [30]. Figure 4 plays a central role in this synopsis, as it possesses important clues on several aspects of CAVD pathophysiology and progression. During EMT, cells on the endocardial side of the primitive heart tube undergo a phenotypic switch from a endothelial-like phenotype to a mesenchymal-like phenotype [31]. These cells migrate into the cardiac jelly portion of the tube, where they form the cardiac cushions which are essential for, among other things, OFT formation [32]. Many transcription factors and signaling pathways have been linked to EMT, as well as neural crest (NC) cell migration to the distal cushions [32]. In absence of NC cells, OFT septation will not occur [33,34]. After fulfilling their role in septation, most NC cells go into apoptosis [35]. Their further role beyond this developmental stage remains largely unknown. Some signaling pathways that play a crucial role during heart valve development include Notch, transforming growth factor beta (TGF-β), vascular endothelial growth factor (VEGF), and Wnt/beta–catenin pathways [15]. Besides signaling pathways, transcription factors expressed in the endocardial cushions represent progenitors of AV leaflets, such a Tbx20, Msx1, Msx2, and Twist1, as well as ECM protein regulators, such as Sox9 and NFATc1 [36]. There has recently been interest in EMT, as it has been shown that adult cardiovascular patients also experience similar transitions when presenting with degenerative diseases of their cardiovascular system [31,37].

### Timing/Expression of Transcription Factors and Deficient Aortic Valve Formation

Valvulogenisis regulatory systems are similar in AVC and OFT development and AVC endocardial cushion formation precedes OFT endocardial cushion formation by one day [15,38]. Given the presumed similarities, the larger AVC endocardial cushions in mouse or chick embryos, and the fact that examination of the OFT cushions is complicated due to the presence of NC derived progenitors (responsible for the formation of the aorto-pulmonary septum), many research extrapolates results from the AVC to the OFT regulatory system [39]. This assumption holds true and has been discussed elsewhere [38,40,41]; here, we highlight some of the pathways that have a more OFT specificity.

Cardiac progenitor cells of the second heart field give rise to several cell lines that play a role during formation of the aortic outflow tract, mediated by Nkx2.5, vascular smooth muscle cells (SMC), endocardial cushion cells, and OFT myocardium [42,43]. Secondary heart field deficiencies preferentially compromise semilunar valve (but not atrioventricular valve) defects that are related to defects in the formation of the OFT [15,41]. Next to that, vascular SMCs of the aortic root originate from these progenitor cells deriving from the second heart field as well as NCC; in contrast, in the ascending aorta and aortic arch, these cells form solely out of neural crest cells [44]. Multiple cell lines and signaling systems are involved in AV and ascending aortic formation; defects in these pathways may induce malformations of the AV, such as BAV. Despite the vast importance of Nkx2.5 in secondary heart field development and the fact that this homeodomain factor is the most commonly mutated single gene in congenital heart disease (CHD), many of its actions remain to be elucidated [45].

**Figure 5:**
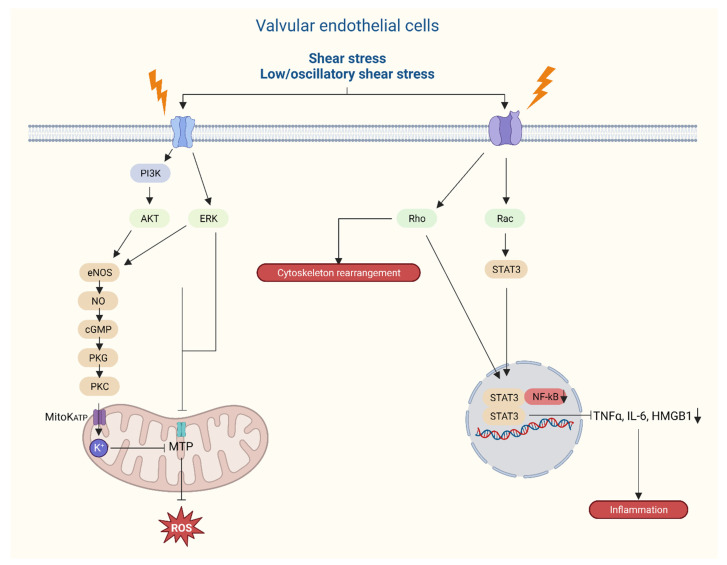
Graphical representation of intracellular responses of aortic valvular endothelial cells following external mechanical stress on the aortic valve leaflets.

The TGF-β superfamily consists of BMPs (BMP2-7) and TGF-β in the embryonic heart where BMPs are responsible for the promotion of endocardial cushion growth. Next to that, BMP, Notch, and TGF-β promote EMT, cell invasion into the cardiac cushions, and remodeling of the valves [23,38,46]. Furthermore, TGF-β has been linked to activation of VICs and their transformation into myofibroblasts in the adult valve [47]. Importantly, Notch signaling disruption markedly decreased the Snail transcription factor responsible for the initial steps in EMT and cushion development [48], placing the Snail pathway at the center of valve development. Notch 1, 2, and 4 receptors and their ligands (Jag 1/2 and Dll4) are specifically expressed in the OFT and its cushions [49,50,51], where inactivation of this pathway leads to a multitude of CHD including BAV [52,53,54]. From a hemodynamical point of view, Notch and downstream pathways have also been linked to a process called valve polarity, distinguishing the lamina fibrosa from the flow side of the valve [15,52] and allowing for valve leaflet maturation [55] (Figure 3). However, this is not yet fully established; reports emphasize the role of Notch signaling in altered shear stress possibly promoting valve stratification and even calcification [51,56] (Figure 4 and Figure 5). A recently published study pointed out the role of the MIB1 gene, an essential regulator for Notch ligands signaling, in the pathophysiology of non-syndromic BAV [57]. Future research should further investigate its potential as a treatment target, along with other genes linked to BAV formation, such as Jag1 [57,58].

In patients with AVD, findings at a transcriptional level may provide clues for innovative treatment strategies. Multi-omics techniques, including proteomics, especially when applied on a population level, aid in understanding the origins and mechanisms of valvar (patho)physiology. For example, studies in the past five years have identified molecular regulatory networks in CAVD that aid in the search for therapeutic targets [59], as well as proteins that were elevated years before CAVD onset, and may be linked to atherosclerotic coronary disease [60]. More recently, using transcriptomic analysis, biological pathways of cardiac ageing, inflammation, and chondrocyte development—through which lipoproteins may cause CAVD—were identified [61]. The metabolic profile of healthy versus diseased cardiac valves have also been mapped using these techniques [62]. The application of such novel methods on failing valve substitutes meets great interest as it may provide valuable insights in the mechanistic basis of valve deterioration, be it native or replaced [63], and possibly its prediction and prevention.

Genome-wide association studies furthermore suggest a role for transcription factors with important embryological functions in the development of AS [64] and bicuspid AS [65], which may help prioritize gene and pathway targets for medical CAVD therapy.

## 4. Anatomy and Hemodynamics

Heart valves open and close around 100,000 times a day, adding up to around 3 billion cycles in a 75-year lifespan, and are subject to a variety of stresses. For many years, the AV has been seen as a “static” structure responsible for unidirectional flow of blood coming from the LV and aiding in coronary perfusion. However, evolving insights shifted our perception of the AV and is now seen as functionally complex regulatory system necessary for optimal mechano-biological coupling of the heart.

### 4.1. The Aortic Valve and Root

Located in the middle of the heart, the AV is commonly referred to as the center of the heart. This is likely no coincidence, as it has several advantages for this particular valve that is subject to the highest levels of pressure and shear stress of all heart valves in a physiological situation [66]. Supported by the fibrous skeleton of the heart and atrioventricular valves, the AV is able to transduce mechanical stress like no other valve, as evidenced by its continuity with the mitral valve (MV) [67]. The pulmonary valve has no fibrous support to it, as it was pushed upwards by the underlying infundibulum, a freestanding rim of muscle seated on the right ventricle and septum directly below the pulmonary leaflets, separating the two right-sided valves during OFT formation [16]. Infundibular muscle, in its turn, is not seen on the left side, where the mitro-aortic fibrous continuity connects the two left-sided valves [68,69]. The AV apparatus was previously described as “a tale of dynamism and crosstalk” by Yacoub [67], which is also underpinned by several treatment modalities respecting the morphology and function of native roots, i.e., the Ross [13], remodeling [70], and reimplantation operations [71].

Throughout evolution, functionality has clearly dictated its morphological counterparts and, as such, the AV can be divided into several functional units (annulus, cusps, sinuses of Valsalva, and sinotubular junction), accumulating into one biomechanical unit. The geometrical, crown-shaped structure of the semilunar valves allows for optimal responsiveness and efficacy during the cardiac cycle as different forces are exerted on the valves [12,72]. Next to that, the precise shape and building plan of the leaflets (including the nodules of Arantius) allow for competent seal and force distribution so that the AV remains competent throughout life. Microarchitectural and geometrical changes may therefore result in biomechanical dysfunction and lead to valvular disease [72], through the pathways illustrated in Figure 4 and Figure 5.

### 4.2. Valvular Fluid Dynamics

**Figure 6:**
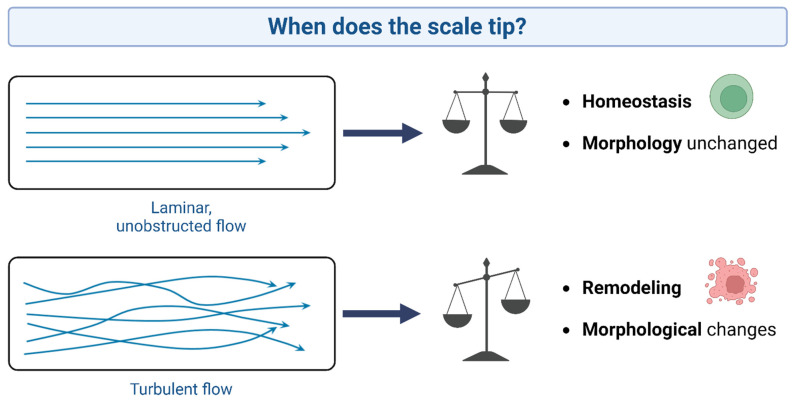
The concept of laminar and turbulent flow and their implications for aortic valve remodeling.

A complex interplay between the cardiac cycle, AV biomechanics, transvalvular hemodynamics, and the compliant properties of the aorta all represent different mechanical stresses that act individually or combined to exert a physiological response through the cellular components of the AV (Figure 6).

The AV is embedded in a crown-shaped annulus [68,69], providing optimal support to the AV under high pressures and dynamic flow patterns. The opening of the AV is a harmonized process and this is crucial to guarantee unimpeded blood flow [73]. Coordinated and competent opening of the valve is essential in decreasing afterload and, therefore, ventricular systolic workload. Nonetheless, alterations in mechanical stress or blood flow near the AV may activate several pathways that in turn may lead to situations in which function deteriorates, as a result of which the LV is exposed to pressure (e.g., in case of AS) or volume (e.g., in case of AR) overload. The opening of the AV lasts for about 330 ms at a heart rate of 70 bpm, where blood rapidly accelerates through the valve, reaching a peak velocity of 1.2 m/s [74,75]. The ensuing deceleration causes a pressure gradient of only several millimeters of mercury with preferential flow at the center of the aorta and low momentum fluid near the aortic wall, thereby causing flow reversal at the sinus regions [74,76,77]. As such, vortices of blood are created by the end of systole, aiding efficient and swift AV closure with an estimated volume (closing volume) to be less than 1% [74,78].

In order to aid fluid dynamics and dictated by transvalvular pressure, the aortic annulus changes shape during cardiac cycle, albeit in an asymmetric fashion, with the greatest expansion during isovolumetric contraction at the left coronary cusp annular region in respect to the non-coronary cusp annular region. Due to its morphological anchoring and continuity with the MV at the site of the non-coronary cusp [68,69], annular stability is provided and allows for energetic transfer from the MV. Next to that, the physiological consequence of this anatomical feature translates into a circumferential increase in diameter at the commissural level, which is proportional to the end-diastolic volume [79]. This biomechanical behavior—where the annulus reaches its minimum size at the end of systole and maximizes in size at the end of diastole [75,78,79]—is a teleonomic hallmark of evolutionary hemodynamics in all mammals. Systolic workload of the ventricle should be as low as possible and, by anticipating the accommodation of each stroke volume, transvalvular hemodynamics are optimized [79], thereby minimizing the possibility of turbulent damage to the valvular cusps. Furthermore, the compliant properties of the surrounding aorta are of vital importance to facilitate ejection as, from a physiological perspective, energetics from cyclic LV contraction need to be addressed in order to provide a continuous flow and pressure downstream in the arterioles [80]. This allows for optimal ventriculo–arterial coupling, and the vertical motion of the aortic root during the heart cycle is important for absorbing stress. In such, both the annulus and the ascending aorta expand to dampen the pressure and flow during systole. This so-called Windkessel effect allows for better energetics where a portion of stroke volume is temporarily maintained by the expanding aorta and later propelled into the circulation by the recoil of the elastic aortic wall [81]. Next to that, another compliancy mechanism takes place regarding the topographical anatomy of the aortic root as it sits at an angle of around 16 degrees to posterior and the left (angle between basal and commissural planes) during diastole. During systole, an alignment of the LV outflow tract (LVOT) and the aorta takes place reducing this angle to around 7 degrees, thereby straightening the tube and thus aiding ejection [12,77,80].

It might be clear that the underlying mechanobiology of the AV is very complex and no sole intervention can preserve all its aspects. Prosthetic valves in the aortic position fix the annulus, are intrinsically obstructive and therefore associated with suboptimal fluid dynamics through the OFT [82]. Even a mild gradient over the valve may have major implications in the long run [5,83], although not directly life-threatening. Image a large closed system, e.g., a bowl, filled with water; should you have a large opening at the bottom, blood will flow out seamlessly, but if it has a small opening, pressure must be increased to maintain equal flow over the defect. The same holds true for AS; the smaller the opening, the more difficult it is for blood to flow out under the same workload. To increase flow, one should increase pressure (workload) before the stenosis or increase the diameter of the opening. Consequently, the velocity of the fluid through the opening has to increase to achieve equal flow, which is in accordance with Bernoulli’s law [84].

In this context, a lifetime of suboptimal gradients and loss of root dynamics, as seen after mechanical or bioprosthetic valve replacement [82], will undoubtedly translate to a higher ventricular workload [85]. In patients undergoing AVR, small reductions in mean transvalvular gradients are associated with significant reductions in heart failure [85]. In such, the Ross procedure, which replaces the diseased AV with the native, living pulmonary valve, enables natural gradients and hemodynamics, coupled with optimal coronary perfusion and ventricular mass regression [86,87]. Physiologic flow patterns and low wall shear stresses after the Ross procedure (full root technique) and valve-sparing root replacement—whether combined with reconstruction of neosinuses or not—are major benefits of these reconstructive approaches [88]. With the current discussions on the lifetime approach to patients with AVD, this holds significant importance.

## 5. Biomechanics and Cellular Responses

**Figure 7:**
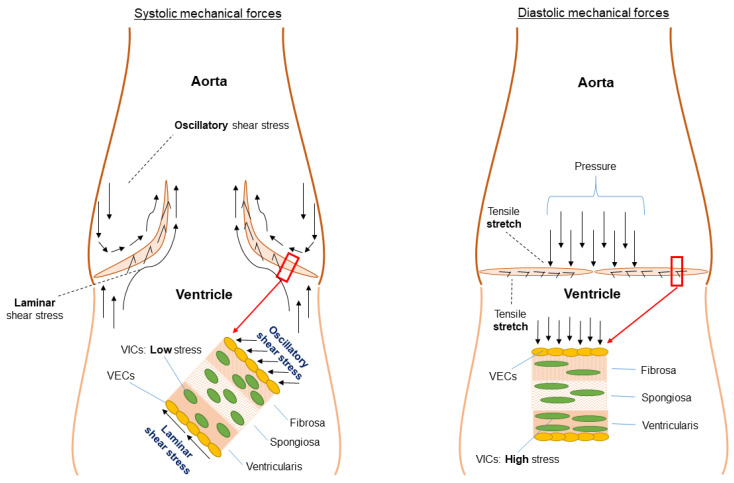
Mechanical stimuli experienced by valvular endothelial cells as well as valvular interstitial cells (fibrosa layer). Adapted/modified from Balachandran et al., 2011 [74], distributed under the CC BY 3.0 DEED Attribution 3.0: Unreported license.

The opening of the AV should be atraumatic as well as symmetric to ensure retained morphology of the apparatus in the long run. Any failure to comply with these terms, i.e., in situations with non-physiological pressures, resistance, or volumes, leads to the activation of second messenger pathways based on mechanical stimuli such as stretch, shear, and transvalvular pressure (Figure 7). Mechanotransduction is the translation of mechanical processes to biological signals, which also affects the aortic valve and root. In a normally functioning AV, the ultimate goal is to efficiently transfer these mechanical stimuli into a well-orchestrated cascade of complex signals. This is reportedly regulated through the cooperative action of valve endothelial cells and VICs [8]. Intrinsic nerve networks are likely responsible for part of these adaptations by regulating synthesis, contraction, repair, and homeostasis within the valve, although evidence for this is not abundantly available.

### 5.1. Functional Morphology and Mechanical Stimuli

Several studies have already highlighted the intricate relationship between hemodynamics and the mechanical microenvironment [77,80,89,90], corroborating the fact that AV degradation is not a passive process. The active interplay between hemodynamics and valvular biomechanical function is attributed to a dense and highly organized ECM network and is defined by the building plan of the leaflets. Different layers are specifically built to cope with different mechanical stresses such as leaflet strain, laminar shear stress, oscillatory shear stress, and pressure as evidenced by the ECM composition of the leaflets that are packed with interstitial cells for structural stability, collagen, elastic fibers, proteoglycans, and glycosaminoglycans, which are lined on both sides with endothelial cells.

Endothelium forms a monolayer of cells, providing a barrier function for the blood and the underlying cells and are the first to be exposed to shear stress (Figure 4 and Figure 7). Based on a bulk of endothelial function research throughout the body, it is rather peculiar that it took quite some time to place endothelial dysfunction at the basis of valvular degeneration [91]. Studies on functional properties of AV endothelium show unique properties compared to other vascular endothelium where the alignment of endothelial cells with regard to the orientation of flow form the most striking difference [92,93]. AV endothelial cells show a perpendicular alignment to flow, a process that is also present in studies using valvular endothelial cells without the presence of an aligned substrate [12,92]. This particular alignment was shown to be dependent on cytoskeletal reorientation; however, it is stimulated by specific endothelial derived mechanotransduction pathways and differential gene expression. Next to that, compared to vascular endothelial cells, the valvular endothelium shows a higher proliferative rate [94] and location of the valvular endothelium (ventricular or aortic side) also seems to play a role in pathway activation. Specific biomechanical profiles and types of shear on the aortic side lead to higher levels of calcification-associated gene and BMP-4 expression; meanwhile, on the ventricular side, inflammation-associated gene expression together with BMP-4 expression are upregulated [93,95,96]. Importantly, different shear stresses are exerted onto the two sides of the AV leaflets, where the aortic side is exposed to interrupted low shear stress as compared to the high-shear-stress ventricular side, with a peak of 70 dynes/m^2^ [66]. Those differences in flow patterns on either sides of the valve leaflets are sensed by the glycocalyx activating signal pathways thereby releasing endothelium-derived vasoactive substances, such as nitric oxide, that play a role in valvular stiffness [9]. Endothelial dysfunction, initiated by lipid deposition, inflammation, mechanical stimuli, and other risk factors—e.g., smoking—produces reactive molecules called reactive oxygen species (ROS) [29], and stimulates a cascade of signaling molecules through valvular interstitial cells (VICs), including TGF-β, interleukin-6, TNF, and BMP-2 [64,97]. The accumulation of these ROS induces several ROS-mediated mechanisms that, in turn, stimulate calcification, mineralization, apoptosis, and osteogenesis, clinically encountered as CAVD (Figure 4). This figure captures key aspects of the risk factors, origins, and actionable targets of AVD.

VICs are highly plastic cells that can alter phenotype, form the dominant AV cell type, and play a crucial role in architectural maintenance and biomechanical functionality of the valve [98]. Optimal biomechanics can be attributed to the ECM as it functions as an integrator between form and function and provides several signaling molecules.

In healthy adults, extracellular homeostasis is regulated by these interstitial cells and mediate valvular remodeling through a balanced secretion of matrix degradation enzymes, including matrix metalloproteinases (MMPs) and their inhibitors (TIMPs), and deposition of structural ECM components within the layers [91], which also play a role in thoracic aortic aneurysm formation [99]. VICs and inflammatory cells stimulate expression of MMP1,2,9 and cathepsins, resulting in abnormal ECM remodeling, which lies at the basis of valvular deterioration [93,98,100]. Deterioration of the valve is based on activated MMP’s and cathepsins degrading collagen and elastin with ensuing pro-inflammatory response, leading to calcification [101]. Furthermore, VICs secrete ECM components such as hyaluronan and collagen, which will deposit in a disorganized fashion, thereby altering valvular stiffness and biomechanical profile; this is aggravated by a VIC transition to osteoblast-like cells [102]. It may be clear that both altered shear stresses and the whole complex interaction between endothelial (dys)function and the ECM will have their effect on the valvular phenotype, resulting in inflammation, degeneration, and calcification.

### 5.2. Calcific Aortic Valve Disease

The macroscopic pathologic anatomy of aortic sclerosis is characterized by nodular calcification and leaflet thickening, which impair the motion—and thereby the function—of the valve. In severe cases, this may contribute to a reduced orifice area, clinically known as AS [103]. Microarchitectural hallmarks of such aortic valve sclerosis include the invasion of inflammatory cells, the induction of fibrosis, and the formation of osteogenic cells, contributing to inflammation and ossification [103,104], indicating that this is an active process. These valvar cells undergo a phenotypic switch or enter apoptotic pathways, driven by increased shear stress, producing ROS, ECM stiffness, and the presence of signaling molecules such as TFG-β1 [102,105].

Portrayed in Figure 4, CAVD is governed by important and unalterable risk factors, such as BAV morphology and patient age. An additional risk is posed by elevated blood pressure, elevated plasma lipoprotein(a) levels, and the presence of diabetes mellitus or obesity—i.e., truly modifiable factors.

Several examples of potential therapeutic targets contributing to CAVD initiation include 3-hydroxy-3-methylglutaryl-coenzyme A (HMG-CoA) reductase, low-density lipoprotein, lipoprotein(a), angiotensin II, angiotensin-converting enzyme, and matrix metalloproteinases [6,106,107,108]. Key studies support a principal, and perhaps causal, role for lipoprotein(a) in CAVD [107,109] and its progression [110]. The effects of statins and angiotensin-converting enzyme inhibition on CAVD progression have been vigorously studied, but the clinical results of such therapies have been variable [108,111]. Administration of statins in asymptomatic AVD patients without risk factors, at this time, should be avoided, as they may induce new risk factors such as diabetes [112,113]. Toll-like receptors (TLRs) function at the interface between tissue repair and innate immunity pathways [114] and it was very recently shown that the TLR3 pathway is an evolutionarily conserved pathway that governs CAVD later in life [114]. Several clinical trials targeting calcium metabolic pathways are ongoing [115].

There have been seminal genetic and molecular studies that have claimed the WNT–β-catenin, Notch, and MYOCD pathways to be involved in the control and commitment of heart valve cells to a fibrocalcific lineage [116]. The activation of these pathways, also involved in the varying embryological steps of AV formation, may contribute to CAVD. The endothelial activation of second messenger pathways related to inflammation, metabolism, and bone formation, instigating this fibrocalcific lineage of the valvular cells, have been shown to lie at the basis of CAVD (Figure 4). Furthermore, circulating osteoprogenitors, likely arising from the bone marrow, that are recruited and are capable of creating a bone-like microenvironment, contribute to valve ossification [117,118,119]. Indeed, the effects of such pathways of valvar degeneration were described more than a decade ago. The next step is the identification of actionable targets based on these hypotheses, which is the subject of extensive ongoing studies [116].

With our increasing understanding of regulatory cellular and genetic pathways, therapeutic targets may be identified and further investigated in a (pre)clinical setting. The yet-unmet potential of these targets is to be elucidated by research focusing on cellular mechanisms of these drugs.

### 5.3. Adaptive Remodeling

To couple the aforementioned concepts to clinical practice, the adaptive capabilities of the pulmonary autograft (Ross procedure) are used to demonstrate this. Prosthetic valve replacement may not sufficiently reproduce biology by restoring native valve function; meanwhile, the Ross procedure allows for a living, dynamic substitute, even showing growth abilities in children [120,121]. It has been correctly postulated that the pulmonary autograft possesses the ability to adapt to the systemic environment by a phenotypic switch to an aortic phenotype after the Ross procedure, as the pulmonary and aortic roots share a common embryological genesis—the conotruncus [122]. However, neural crest cells, compromised in congenital AS, are less commonly seen in pulmonary than in aortic roots in murine embryological studies [123]. Such findings may explain why anatomic pulmonary valve anomalies, impeding use of a pulmonary autograft, are rare (incidence: 0.1%) and usually associated with other heart defects [124]. Explanted autografts are populated by viable valvular interstitial and endothelial cells after several years [125]. This process is called adaptive remodeling and is facilitated by gradually exposing the autograft to the systemic environment—for example, through systolic blood pressure management below 110 mmHg during the first 6–12 months postoperatively [126]. These adaptive capabilities all result from the concepts of mechanotransduction and activation of adaptive messenger pathways, as described here. This is also suggested by expression of the gene EphrinB2 in autograft endothelium after the Ross procedure, which is a biomarker of left-sided, but not right-sided, heart valve endothelium. This induced expression of EphrinB2 stimulates ECM remodeling, leading to increased production of smooth muscle actin [125,127]. In CAVD, as well as adaptive autograft remodeling, there exists a desire to understand whether there is a point of no return—i.e., a tipping point—in native tissue’s ability for adaptation, after which a state of maladaptation and disease is produced. Further insights into biomarkers, leaflet stress and innovative imaging techniques may aid in objectifying a patient’s physiological reserve, identifying such thresholds [128], and moving towards personalized medicine.

Surgical modifications—i.e., autograft reinforcement [126,129,130]—and adjuncts to conventional postoperative management strategies—i.e., beta-blocker-driven blood pressure regulation—have been proposed, some to promote adaptive remodeling and all to prevent autograft dilatation. Given the concepts put forward in this review, it appears salient to realize that no technique is perfect, all choices will affect outcomes, and there should be a balance between the support provided and dynamism preserved. 

From a biomechanical standpoint, the suboptimal alignment of the components of the AV will increase shear (oscillatory) stress on the AV leaflets and proximal aorta in addition to the systolic loss of energy in the LV [131]. As an example, chronic oscillatory stress of the different component parts is thought to cause premature deterioration after the subcoronary Ross operation [131]. Adjuncts performed to the total root technique of the Ross procedure should thoughtfully balance support with maintenance of valve dynamism.

## 6. (Surgical) Treatment

The embryology, transcription, fluid dynamics, mechanobiology, and cellular pathways involved in AVD have been the subject of extensive study. Integration of these concepts into the clinical decision-making process is complex given the widely differing levels of information provided through the learnings in these varying fields of interest.

### 6.1. Evidence-Based Medicine

Evidence-based medicine has previously been elegantly illustrated as a three-legged stool, which exemplifies that the best available evidence is just one leg of this stool [132]. The other two legs, physician’s skills and expertise and patient values and expectations, cannot be left out of the equation during clinical decisions. In other words, evidence-based medicine is not “cookbook” medicine [132,133]. Prosthetic valve selection still carries several challenges pertaining to a lack of robust evidence and widely varying patient values and expectations between individuals. Current options for AVR include bioprosthetic AVR (surgically or transcatheter), mechanical AVR, the Ross procedure, and homograft AVR [134,135]. Bioprostheses are commercially available and do not require lifelong anticoagulation, but they exhibit limited durability [134]. Mechanical prostheses are designed to last a lifetime, but they produce a ticking sound and have a thrombogenic surface, therefore requiring lifelong anticoagulation, translating to increased bleeding and thrombo-embolism hazards [136]. Homografts come from human donor tissue and do not require anticoagulation, but they show premature calcifications and early failure [137,138]. The Ross procedure is the only living aortic valve substitute available [13], translating to optimal hemodynamics, requiring no anticoagulation, and having excellent long-term outcomes in experienced hands [137,139]. However, it transforms single-valve disease to double-valve disease and is technically demanding [140]. The unique benefits and drawbacks of all substitutes become immediately clear, but it remains a challenge to implement this into the decision-making process. Prostheses still cannot reproduce the complexity of human nature, and Donald Ross in 1967 correctly postulated that a living valve substitute was necessary to ensure longevity of a valve substitute [13]. The Ross procedure has known times of little acceptance and adoption in clinical practice, with a trough around the year 2010 [141]. Previous data on the long-term outcomes of this operation have been discouraging in some instances, but novel, contemporary data show excellent long-term results with survival that is comparable to the matched general population [137,139,142]. Besides anticoagulation avoidance, low rates of endocarditis have been reported after the Ross procedure [139,142], which should be considered when contemplating the optimal lifetime management of AVD patients. Insights into the technical success factors and improvements in patient selection have led to a standardized operation that is reproducible. The growing data favoring the Ross procedure support a reevaluation of the guidelines for the treatment of AVD, but the increased enthusiasm for the Ross procedure should be carefully balanced with its increased technical complexity by concentrating them in Ross centers of excellence [139,143,144].

### 6.2. Clinical Decision Making

Decision making in AVD is complex and entails much more than the available evidence and the clinical state of the patient. There is an ongoing shift toward tailoring treatment to the individual patient’s needs and circumstances, taking patient values and goals into account, as well as the short- and long-term advantages and disadvantages of different treatment options (survival and complications, quality of life). The lifetime aspect of AVD adds another dimension to the decision-making process, as decisions made now will undoubtedly influence later decisions and outcome. This makes one appreciate the potential harm of avoiding risk in the short term, since this may produce higher risks in the long term [145]. So, individuals do not only benefit from tailored treatment options in the present day, but also in the future, all while taking into account patient values. Hence, one should aim for strategic planning of interventions over a lifetime, bearing in mind the options for a second and perhaps third intervention during index procedure planning, in an informed, shared decision-making process together with the patient.

We can acknowledge that no valve substitute or treatment solution is perfect. Circling back to the basis of this review, on the other hand, we can now appreciate that the Ross procedure comes closest to an ideal solution in terms of its biomechanics, embryological origin, anatomy/geometry, gene transcription and cellular responses, although the longitudinal functional decline hazards of the autograft can be improved. Novel treatment options are direly needed to meet the needs of patients with AVD. For the future, tissue engineering of heart valves (TEHV), to be regarded a byproduct of the Ross procedure, meets great interest. TEHV can produce a living valve able to emulate the sophisticated functions of a native valve [146,147]. The concept of in situ regeneration, which uses the microenvironment as a natural bioreactor, has produced encouraging results in recent years [148,149,150]. Cellular repopulation of an acellular scaffold has recently been successfully shown in a sheep model [150], with endothelial cells and nerves connecting with contractile cells and blood vessels, just like in a native valve [150].

A method able to simulate individual patient lives and generate disease-, age-, and sex-specific estimates of patient outcome, microsimulation models may fulfill a role in objectifying these different lifetime treatment pathways and their outcomes [120,134,136,151,152]. For the future, these models should become more patient-tailored and its potential for modeling the effect of certain treatment decisions and sequential treatments over a lifetime should be explored.

## 7. Conclusions and Directions

The mechanistic basis of AVD, and in particular aortic sclerosis, is that it is an active process, modulated by endothelial and interstitial valvar cells, that is also governed by the biomechanical environment of the valve. Techniques such as proteomics represent a promising avenue to enhance our understanding of the mechanistic basis of CAVD, and its prevention. In practice, treatment decision making for AVD is complex and is based on much more than scientific evidence, be it cellular or clinical. The interplay seen in a healthy, native aortic valve is a perfectly synchronized, almost magical, dynamic process and the undeniable clinical benefits of a living valve have been repetitively addressed.

The Ross procedure provides AVD patients with a viable therapeutic option integrated into the functional aortic root unit, also demonstrating an optimal postoperative quality of life and a favorable life expectancy. This review furthermore identified gaps that invite research on AVD (mechano)biology, proteomics, and (epi)genetics to identify cellular therapeutic targets and biomarkers of maladaptation.

## Data Availability

No new data were created or analyzed in this study. Data sharing is not applicable to this article.
